# Inhibitory Effects of Nobiletin on Voltage-Gated Na^+^ Channel in Rat Ventricular Myocytes Based on Electrophysiological Analysis and Molecular Docking Method

**DOI:** 10.3390/ijms232315175

**Published:** 2022-12-02

**Authors:** Youwei Gu, Jieru Wang, Mengting Li, Fei Zhong, Jie Xiang, Zhengxin Xu

**Affiliations:** 1 Department of Pharmacology, School of Medicine, Yangzhou University, Yangzhou 225009, China; 2 Jiangsu Co-Innovation Center for Prevention and Control of Important Animal Infectious Diseases and Zoonoses, Yangzhou 225009, China; 3 Jingsu Key Laboratory of Integrated Traditional Chinese and Western Medicine for Prevention and Treatment of Senile Diseases, Yangzhou 225001, China; 4 Jiangsu Key Laboratory of Experimental & Translational Non-Coding RNA Research, Yangzhou 225009, China; 5 Yeda Institute of Gene and Cell Therapy, Taizhou 318000, China

**Keywords:** nobiletin, arrhythmia, Na_V_1.5 channel, ventricular myocytes, amiodarone, aconitine, molecular docking simulation

## Abstract

Nobiletin (NOB) has attracted much attention owing to its outstanding bioactivities. This study aimed to investigate its anti-arrhythmic effect through electrophysiological and molecular docking studies. We assessed the anti-arrhythmic effects of NOB using aconitine-induced ventricular arrhythmia in a rat model and the electrophysiological effects of NOB on rat cardiomyocytes utilizing whole-cell patch-clamp techniques. Moreover, we investigated the binding characters of NOB with rNav1.5, rNav1.5/QQQ, and hNa_V_1.5 via docking analysis, comparing them with amiodarone and aconitine. NOB pretreatment delayed susceptibility to ventricular premature and ventricular tachycardia and decreased the incidence of fatal ventricular fibrillation. Whole-cell patch-clamp assays demonstrated that the peak current density of the voltage-gated Na^+^ channel current was reversibly reduced by NOB in a concentration-dependent manner. The steady-state activation and recovery curves were shifted in the positive direction along the voltage axis, and the steady-state inactivation curve was shifted in the negative direction along the voltage axis, as shown by gating kinetics. The molecular docking study showed NOB formed a π-π stacking interaction with rNav1.5 and rNav1.5/QQQ upon Phe-1762, which is the homolog to Phe-1760 in hNa_V_1.5 and plays an important role in antiarrhythmic action This study reveals that NOB may act as a class I sodium channel anti-arrhythmia agent.

## 1. Introduction

Cardiac arrhythmias are one of the most common cardiovascular diseases, and cardiac arrest caused by organic heart disease with arrhythmias is a leading contributor to sudden cardiac death [1,2]. The prognosis and survival rates of patients who suffer from ventricular tachycardia or ventricular fibrillation remain unsatisfactory [3]. The resting potential of cell membranes is frequently disturbed in ventricular arrhythmias and organic heart disease, which leads to the disorder of ionic channel currents. In addition, malignant cardiovascular diseases may be caused by aberrant changes in the ion channel expression or gating kinetics in cardiomyocytes [4,5].

Ion channels are essential building blocks of various action potential currents in cardiac myocytes, and voltage-gated ion channels are crucial for understanding their structures, distributions, interactions, and functions [6]. The Na^+^ channel current (*I*_Na_), which begins with the activation of voltage-gated Na^+^ channels encoded by *SCN5A*, is the most important part of the 0 phase of the entire action potential [7]. Decreased function of the Na_V_1.5 often impedes inactivation prolongation, resulting in Brugada syndrome (BrS) and cardiac conduction syndrome. The abnormal elevated function of Na_V_1.5 in cardiac myocytes may result in type 3 long QT syndrome (LQT3) and sudden infant death syndrome, both of which are considered pro-arrhythmic [8]. So far, 154 and 230 missense mutations associated with LQT3 and BrS have been discovered, respectively [9]. By regulating the expression and function of the sodium channel, the peak intensity and kinetic gating characteristics of the *I*_Na_ can be adjusted, allowing for the therapy of myocardial remodeling, conduction block, and other cardiac disorders.

Nobiletin (NOB), a polymethoxylated flavonoid, has exhibited a wide range of biological effects owing to its unique structure character and good lipid solubility [10,11]. Researchers have recently discovered that NOB, as an inhibitor of the renin–angiotensin system, can reverse left ventricular dysfunction and remodeling in two-kidney, one-clip-induced hypertensive rats [12]. What is more, NOB has been studied to exhibit protective effects on cardiovascular dysfunction in diabetic rats by inhibiting the activity of matrix metalloproteinase-2 and matrix metalloproteinase-9 [13]. Moreover, previous studies have shown that NOB plays, at least in part, an essential role in ameliorating cardiac impairment after acute myocardial infarction in rats [14,15]. In diabetic cardiomyopathy, the pathological changes of myocardial cells have often been described as leading to reductions in cell capacitance and the density of Na^+^ ion channels [16]. Furthermore, target prediction by pre-screening network pharmacology analysis identified NOB in Xin-Su-Ning capsules as a possible quality marker against arrhythmia [17].

In this work, we aimed to investigate the potential anti-arrhythmic effects of NOB combining electrophysiological and molecular docking analysis to further reveal its cardioprotective activity. We hypothesized that the activity of NOB is partially due to the inhibition of *I*_Na_ in ventricular myocytes because of direct binding to active sites.

## 2. Results

### 2.1. NOB Alleviated Fatal Ventricular Arrhythmia In Vivo

**Figure 1:**
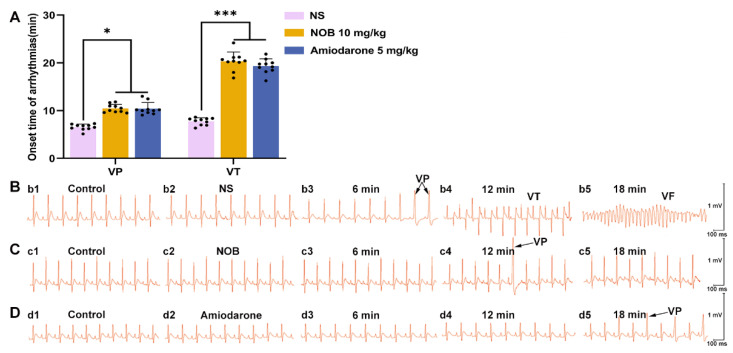
Effects of nobiletin on aconitine-induced arrhythmia in rats. (**A**) Onset time of aconitine-induced ventricular arrhythmia in the normal saline group (Saline, *n* = 10), nobiletin group (NOB, *n* = 10), and amiodarone group (*n* = 10). (**B**) Representative ECG traces at time of onset in the Saline group (chosen from a male rat). (**C**) Representative ECG traces of the NOB group at the same time as the control group (chosen from a male rat). (**D**) Representative ECG traces of the amiodarone group at the same time as the control group (chosen from a female rat). ECG, electrocardiogram; NOB, nobiletin; VP, ventricular premature contraction; VT, ventricular tachycardia; VF, ventricular fibrillation. (*n* = 10, * *p* < 0.05, *** *p* < 0.001 vs. Saline group).

In the current study, a fatal cardiac arrhythmia model was established by administering aconitine intravenously. The onset times of ventricular premature contraction (VP) and ventricular tachycardia (VT), as well as the incidence of ventricular fibrillation (VF), were used to assess the severity of arrhythmia. Pretreatment with NOB postponed the onset of VP and VT and substantially reduced the morbidity of VF (Figure 1 and Table 1). Compared to the normal saline group (Saline group), the therapeutic effects of NOB (10 mg/kg) were similar to those of amiodarone. Overall, NOB significantly alleviated aconitine-induced fatal ventricular arrhythmias.

### 2.2. NOB Inhibited I_Na_ in a Concentration-Dependent Manner

**Figure 2:**
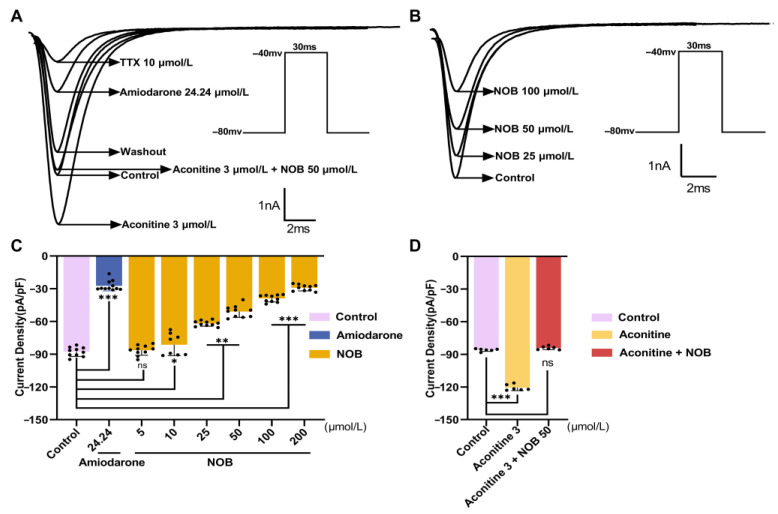
Inhibitory effects of nobiletin (NOB; 25, 50, and 100 μmol/L) and amiodarone on *I*_Na_ in rat ventricular myocytes. (**A**) Effects of tetrodotoxin (TTX), amiodarone, aconitine, and aconitine with NOB on *I*_Na_. (**B**) Original current diagram of NOB on *I*_Na_. (**C**) Effects of different concentrations of NOB and amiodarone on *I*_Na_. Data are expressed as means ± standard deviation (*n* = 10, measured in six rats). (**D**) Effect of aconitine (3 μmol/L) and aconitine (3 μmol/L) with NOB (50 μmol/L) on *I*_Na_ compared to Control group. Data are expressed as means ± standard deviation (*n* = 6, measured in three rats); ns, no significant difference; * *p* < 0.05, ** *p* < 0.01, *** *p* < 0.001 vs. control group.

Tetrodotoxin (TTX, 10 μmol/L) was used as a sodium-channel-specific blocker (Figure 2). The current was mostly restored after removing TTX in the extracellular environment, indicating that the inward current of this stimulation protocol was *I*_Na_. Before evaluating the administration of NOB, the current was maintained at a steady state for 5–10 min. NOB at 5 and 10 μmol/L had no obvious effects on *I*_Na_, compared with the control group (Table 2). Furthermore, 25, 50, and 100 μmol/L NOB substantially inhibited *I*_Na_ in a concentration-dependent manner. Compared with amiodarone, 100 μmol/L NOB had a slightly weaker inhibitory effect. Amounts of 25, 50, and 100 μmol/L NOB and 24.24 μmol/L amiodarone reduced the peak sodium current density, demonstrating inhibitory rates of 29.53 ± 2.90%, 42.04 ± 6.16%, 55.69 ± 3.66% (*n* = 10, *p* < 0.01), and 68.86 ± 5.28% (*n* = 10, *p* < 0.01), respectively, under −40 mV depolarization voltage. In the concentration–response curve, the fractional blockage was plotted against the appropriate concentrations of NOB. The Hill equation matched the curve [18]:

Y = 100/{1 + (IC_50_/X)^HillSlope^} (1)

where Y is the normalized response of the inhibitory rate, and X is the concentration of NOB.

The half maximal inhibitory concentration (IC_50_) of NOB on the peak sodium current was 54.57 μmol/L (49.10–61.46 μmol/L, 95% CI).

### 2.3. Effects of NOB on the Current–Voltage Curve (I–V Curve) of I_Na_

**Figure 3:**
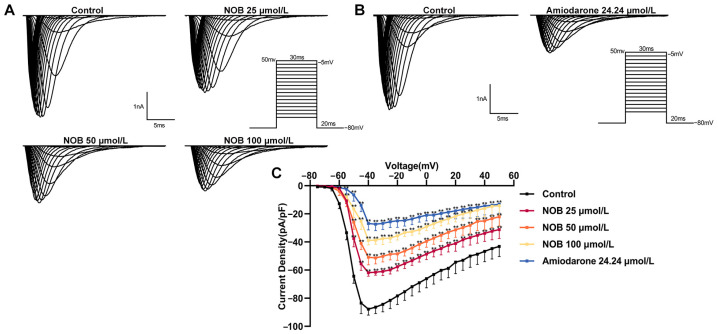
Inhibitory effect of nobiletin (NOB) and amiodarone on the current–voltage (*I*–*V*) curve. (**A**) *I*–*V* trace recorded in the absence or presence of different concentrations of NOB. (**B**) *I*–*V* trace with or without 24.24 μmol/L amiodarone. (**C**) Effects of NOB and amiodarone on *I*_Na_ and upward shift of the *I*–*V* curve. Data are expressed as means ± standard deviation (*n* = 10, measured from six rats); ** *p* < 0.01 vs. Control group.

To explore the effects of NOB on the *I*_Na_ under different voltage conditions, changes in *I*_Na_ produced by the consecutive stimulations were gathered in the absence and presence of the administration of NOB and amiodarone. The results show that 25, 50, and 100 μmol/L NOB and 24.24 μmol/L amiodarone decreased the depolarized current without changing the form or trend of the curve (Figure 3). Moreover, the *I*–*V* curve shifted along the positive axis concentration-dependently. Overall, the current density was reduced under the protocol voltages, especially between applied voltages of −40 mV and −30 mV.

### 2.4. Effects of NOB on the Steady-State I_Na_ Activation Curve

**Figure 4:**
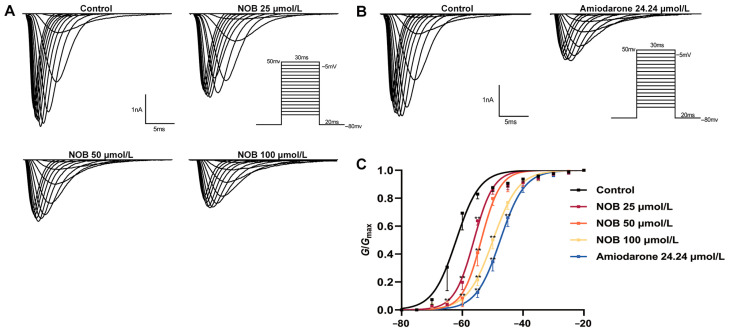
Effects of nobiletin (NOB) and amiodarone on steady-state activation in rat ventricular myocytes. (**A**) Steady-state activation curve in the form of original diagrams with or without 25, 50, and 100 μmol/L NOB. (**B**) With or without 24.24 μmol/L amiodarone. (**C**) Effects of NOB and amiodarone on fitted activation curve. Data are expressed as means ± standard deviation (*n* = 7, measured from four rats); ** *p* < 0.01 vs. Control group.

To investigate the effect of NOB on the activation and opening of sodium channels, the variations in *I*_Na_ with and without the administration of NOB and amiodarone were recorded (Figure 4). The data pertaining to the Na_V_1.5 channel were processed to draw the activation curve fitted by the Boltzmann equation [19]:

*G*/*G*_max_= 1/{1 + exp(*V* − *V*_1/2_)/*κ*} (2)

where *G* is the electric conductance, and *κ* is the slope factor.

The administration of NOB at different concentrations caused the steady-state activation curve to shift positively along the voltage axis, i.e., a depolarization trend, and the half activation voltage (*V*_1/2-ac_) increased. Additionally, 25, 50, and 100 μmol/L NOB and 24.24 μmol/L amiodarone changed the *V*_1/2-ac_ value from −62.00 ± 0.53 to −56.17 ± 0.44, −53.77 ± 0.29, −49.89 ± 0.18 mV (*n* = 7, *p* < 0.01), and −47.52 ± 0.16 mV (*n* = 7, *p* < 0.01), respectively. The administration of NOB therefore suppressed the opening process of the Na^+^ channel and raised the critical value for Na^+^ channel activation, making the channel opening progress more difficult.

### 2.5. Effects of NOB on the I_Na_ Inactivation Curve

**Figure 5:**
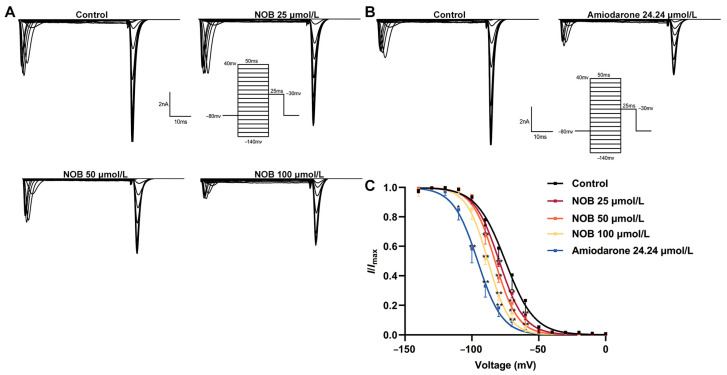
Inhibitory effects of nobiletin (NOB) and amiodarone on the steady-state inactivation curve. (**A**) Inactivation curves recorded in the form of original diagrams under control conditions and 5 min after the addition of 25, 50, and 100 μmol/L NOB. (**B**) Absence or presence of 24.24 μmol/L amiodarone. (**C**) The steady-state inactivation curve of *I*_Na_ obtained by fitting the Boltzmann equation in the presence of different concentrations of NOB and amiodarone. Data are expressed as means ± standard deviation (*n* = 7, measured from four rats); * *p* < 0.05, ** *p* < 0.01 vs. Control group.

To analyze the effects of NOB on the *I*_Na_ inactivation curve, the variation in *I*_Na_ before and after the administration of NOB and amiodarone was elicited using double-pulse stimulation (Figure 5). The inactivation data were fitted using the Boltzmann equation [19]:

*I*/*I*_max_= 1/{1 + exp(*V* − *V*_1/2_)/*κ*} (3)

where *κ* is the slope factor.

The administration of NOB at different concentrations caused the steady inactivation curve to shift negatively along the voltage axis, i.e., a hyperpolarization trend; the half inactivation voltage (*V*_1/2-in_) decreased. Overall, the administration of 25, 50, and 100 μmol/L NOB and 24.24 μmol/L amiodarone reduced the V_1/2-in_ from −75.00 ± 0.57 to −79.59 ± 0.44, −82.72 ± 0.64, −87.94 ± 0.60 (*n* = 7, *p* < 0.01), and −95.69 ± 0.54 mV (*n* = 7, *p* < 0.01), respectively. Moreover, the *κ* value was altered from 8.03 ± 0.53 to 8.55 ± 0.56 (*n* = 7, *p* > 0.05), 9.23 ± 0.47, 9.39 ± 0.39 (*n* = 7, *p* < 0.01), and 10.80 ± 0.5 (*n* = 7, *p* < 0.01), respectively. The results suggest that NOB may have a strong affinity for binding to sodium channels during inactivation, slowing the time-dependent Na_V_1.5 channel inactivation.

### 2.6. Effects of NOB on Recovery Curves after Inactivation of I_Na_

**Figure 6:**
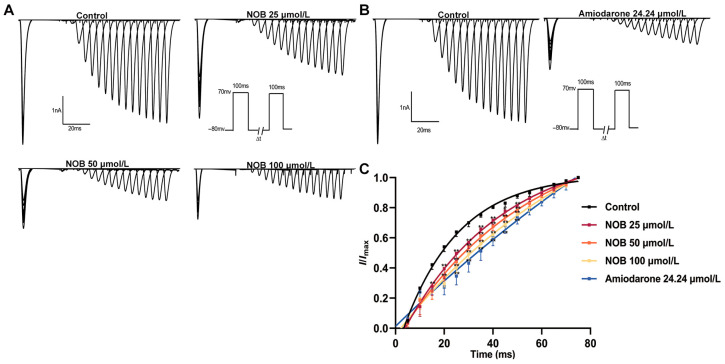
Effects of different concentrations of nobiletin (NOB) and amiodarone on recovery curves after inactivation of *I*_Na_ in rat ventricular myocytes. (**A**) Recovery curves recorded in the form of original diagrams under control conditions and 5 min after the addition of 25, 50, and 100 μmol/L NOB. (**B**) With or without 24.24 μmol/L amiodarone. (**C**) Recovery curve after inactivation in the presence of NOB (25, 50, 100 μmol/L) and 24.24 μmol/L amiodarone. Data are expressed as means ± standard deviation (*n* = 7, measured from four rats); ** *p* < 0.01 vs. Control group.

To study the effects of NOB on the *I*_Na_ recovery curve, the variation in *I*_Na_ was recorded using two-pulse stimulations, before and after the addition of different concentrations of NOB and amiodarone (Figure 6). The recovery data for *I*_Na_ were fitted using the one-phase association equation [19]:

*I*/*I*max = 1 − exp(−*t*/*τ*) (4)

where *t* is the time interval between two pulses, and *τ* is the time constant of reactivation after inactivation.

The recovery curve was positively shifted along the voltage axis following treatment with NOB and amiodarone. The results of the curve showed that 25, 50, and 100 μmol/L NOB and 24.24 μmol/L amiodarone changed the τ value from 21.51 ± 0.95 to 26.75 ± 0.94, 29.16 ± 0.34, 32.04 ± 0.78 (*n* = 7, *p* < 0.01), and 35.48 ± 1.12 ms (*n* = 7, *p* < 0.01), respectively. These results suggest that NOB and amiodarone significantly changed the recovery kinetics of *I*_Na_ after inactivation and procrastinated the process of *I*_Na_ from inactivation to activation. This also indicates that NOB has a considerable affinity for the recovery channel from inactivation to activation.

### 2.7. Molecular Docking Simulation

**Figure 7:**
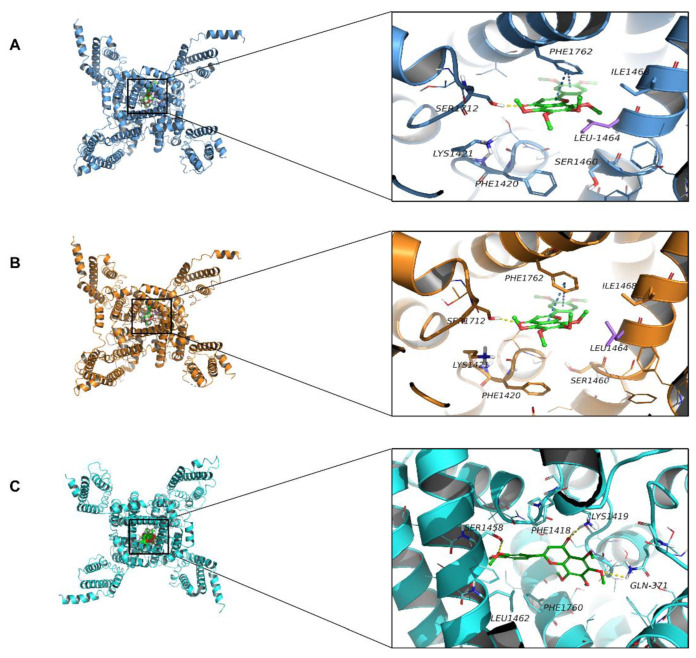
Computer modeling of active sites regarding the potential key target of nobiletin in the active domain of the rat voltage-gated sodium channel (termed rNav1.5) (**A**), open state structure of rNav1.5 (termed rNav1.5/QQQ) (**B**), and human voltage-gated sodium channel (termed hNav1.5) (**C**). The residues of ligand bind domain (LBD) proteins representing hydrogen bonds are depicted and annotated as yellow dotted lines.

The molecular docking method was performed to explore how NOB interacted with the rat voltage-gated sodium channel (termed rNav1.5), the open state structure of rNav1.5 (termed rNav1.5/QQQ), and the human voltage-gated sodium channel (termed hNa_V_1.5) by computer-based docking stimulation (Figure 7, Table 3). The docking analysis between aconitine and amiodarone with hNa_V_1.5 was performed and compared with NOB (Figure S1, Table S2). The docking model predicted that NOB has affinity binding energies of −6.655 and −6.562 kcal/mol to rNav1.5 and rNav1.5/QQQ, respectively. More specifically, NOB formed a π-π stacking interaction, a hydrogen bond with rNav1.5, and rNav1.5/QQQ upon Phe-1762 and Ser-1712 residues, respectively. In addition, there were five hydrophobic contacts that contributed to both the NOB-rNav1.5 and NOB-rNav1.5/QQQ binding, namely, Ile-1468, Leu-1464, Ser-1460, Phe-1420, and Lys-1421 (Figure 7, Table 3). Although the NOB-rNav1.5 and NOB-rNav1.5/QQQ binding exhibited the same intermolecular binding interactions, their binding energy difference was due to the lower hydrophobic binding with Leu-1464 of NOB-rNav1.5/QQQ compared with NOB-rNav1.5 docking. The binding affinity of NOB, aconitine, and amiodarone, had the lowest energy poses, shown by the Gibbs free-energy values (ΔG) as −5.693, −8.889, and −6.465 kcal/mol, respectively (Table 3, Tables S1 and S2), with hNa_V_1.5. Furthermore, the molecular mechanics/generalized Born surface area (MM-GBSA) was used to predict the binding free energies of NOB, aconitine, and amiodarone to the human Na_V_1.5 [20]. NOB, aconitine and amiodarone exhibited low glide energy at −51.71 kcal/mol, −62.91 kcal/mol, and −42.26 kcal/mol against hNa_V_1.5, respectively, where molecular docking simulation found good binding affinity (Table S1). In particular, NOB and aconitine were mainly docked in the transmembrane helices of voltage-sensing domains (VSD) III and IV of hNa_V_1.5, with different types of binding interactions (Figure 7 and Figure S1).

Protein–ligand interaction profiler analysis demonstrated that NOB formed hydrogen bonds with Gln-371, Lys-1419, and Ser-1458, and hydrophobic interaction with Phe-1418, Leu-1462, and Phe-1760 when docking with hNa_V_1.5. Aconitine formed a hydrogen bond with Gln-371 and hydrophobic interactions with Leu-931, Leu-1462, Ser-1458, Phe-1418, Lys-1419, Val-405, and Phe-1760. Although the ΔG of NOB to hNa_V_1.5 was higher than that of aconitine, it could establish more hydrogen bonds with hNa_V_1.5 than the latter. Notably, amiodarone formed cation-π with Phe934, π-π stacking with Phe1760, and hydrophobic interactions with Phe-1418, Lys-1419, Ser-1458, and Leu-1462 when docking with hNa_V_1.5 (Figure S1, Table S2). 

## 3. Discussion

The electrophysiological function of the heart is based on its dynamic ionic balance, which is frequently altered in aging or sick hearts and is regarded as a significant trigger for arrhythmias [21]. Aconitine is a highly cardiotoxic sodium channel agonist that is frequently utilized for modeling purposes in investigations. The mechanism of aconitine-induced arrhythmia relies on its ability to bind to Na_V_1.5 and sodium–calcium exchanger and prolong their openings, which facilitates the entry of Na^+^ into the cell and results in long-lasting depolarization and calcium overload [22,23]. These effects cause automaticity in fast-responding cells and are similar to the abnormally high inward flow of Na^+^ flux that develops in the presence of enhanced triggered activity in ischemic heart diseases [24]. Moreover, aberrant mutations in *SCN5A*, which encodes the Na_V_1.5 channel, can cause a variety of genetic arrhythmia syndromes, including LQT3, Brugada syndrome, progressive cardiac conduction disorder, sudden infant death syndrome, and different cardiac conduction abnormalities [17,25]. Therefore, the regulation of the Na_V_1.5 current and the expression of the encoded Na_V_1.5 channel *SCN5A* are crucial for the treatment of malignant arrhythmia [26]. 

In mammals, the nine subtypes of the human voltage-gated sodium (Na_V_1.1- Na_V_1.9) channels are responsible for the initiation and transmission of electrical impulses in different tissues and Na_V_1.5 is the primary cardiac isoform. The topology of mammalian Na_V_ channels comprised Domain I, Domain II, Domain III, IFM-motif, and Domain IV of the α-subunit, and the β1 and β2 subunits [27]. The sequence alignments of rNav1.5 (Uniprot ID: P15389) and hNa_V_1.5 (Uniprot ID: Q14524) are compared in Figure S2 and exhibit more than 94% similarity. The open state structure of rNav1.5 was achieved by importing the IFM/QQQ mutations to remove the fast inactivation gate [28].

In this study, NOB protected against aconitine-induced ventricular arrhythmias and substantially slowed the onset of lethal ventricular fibrillation, similar to amiodarone. The peak *I*_Na_ was substantially blocked by NOB in vitro with a dependent manner of voltage and concentration. Notably, NOB substantially shifted the activation and recovery curves along the voltage axis positively and the inactivation curve negatively. This suggests that NOB affects the kinetic gating characteristics of Na^+^ channels, accelerating the inactivation and retarding the post-inactivation recovery of Na_V_1.5 channels. In this experiment, for the first time NOB was docked with rNav1.5 and rNav1.5/QQQ. NOB, aconitine, and amiodarone were docked with hNa_V_1.5, and their molecular effects were described. The molecular docking study showed that NOB formed a π-π stacking interaction with rNav1.5 and rNav1.5/QQQ upon Phe-1762, which is the homolog to Phe-1760 in hNa_V_1.5 and plays an important role in antiarrhythmic action [29,30]. The computational modeling results have further verified the patch-clamp results of the inhibitory effects of NOB on Nav1.5 in rat ventricular myocytes in both closed and open states. 

In the molecular docking stimulation, both NOB and aconitine formed hydrogen binding with Gln-371, which provides a reasonable explanation for how NOB and aconitine affected the kinetics of Na^+^ gating in cardiomyocytes. Compared to aconitine, NOB forms additional hydrogen bonds with Lys-1419 and Ser-1458 of hNa_V_1.5. In addition, NOB formed hydrophobic interactions with Phe-1418, Leu-1462, Phe-1760, and aconitine interacted with the Na_V_1.5 channel by hydrophobic bonds such as Leu-931, Leu-1462, Ser-1458, Phe-1418, Lys-1419, Val-405, and Phe-1760. Several studies have demonstrated that residues Gln-371 and Leu-1462 of hNa_V_1.5 exert a vital role in the dynamics of steady-state activation and inactivation [31]. In addition, amiodarone exhibited strong bindings as cation-π and π-π stacking interactions with Phe-934 and Phe-1760, respectively, when docking with hNa_V_1.5. Amiodarone, possessing a stronger interaction with Phe-1760, which is the backbone of the bonding site of Na^+^ with hNa_V_1.5, showed excessive inhibitory activity against Na_V_1.5 [32]. This excessive blockage is closely related to the most commonly reported adverse reactions in clinical application, such as conduction block and bradycardia [33]. It corresponded with the patch-clamp electrophysical results that amiodarone explicated greater inhibitory activity in *I*_Na_ than aconitine and NOB. In summary, NOB inhibited the Na_V_1.5 channel by substantially suppressing the peak *I*_Na_, reducing the channel opening levels, and retarding voltage-dependent channel inactivation and recovery via binding to inactivated channels. Consequently, NOB exhibited an inhibitory impact on the Na^+^ channel in cardiomyocytes. Compared to amiodarone, which is already widely used in clinical practice in the treatment of arrhythmia, the inhibitory effect of NOB on cardiomyocyte Na_V_1.5 was similar but more moderate. Exploring the mechanisms by which NOB (a potential antiarrhythmic drug in this study), amiodarone (the most widely used clinical antiarrhythmic drug), and aconitine (a widely used tool drug for ventricular arrhythmias) bind to Na_V_1.5 by the molecular docking method will help us to gain more understanding of the structure–activity relationship, and to develop novel agents from natural sources for treating ventricular arrhythmias.

The SARS-CoV-2 infection is a high-risk-inducing factor for cardiovascular disease due to severe cytokine storms and systemic inflammatory responses [34,35,36,37,38]. Anti-arrhythmic drugs currently in clinical use, such as amiodarone, which has pulmonary toxicity, and quinidine, which has proven to show a pro-arrhythmia effect, may aggravate the damage to the cardiopulmonary circulatory system [39,40,41,42]. Therefore, the development of low-toxicity and multi-target cardiovascular drugs is also urgently needed [43]. Previous studies have reported that NOB exerts anti-inflammatory, antioxidant, and anti-hyperlipidemic effects to prevent the development of cardiovascular disease [12,14,15,44,45]. We speculate that NOB-rich supplements or other forms of agents may represent promising multi-target therapeutic candidates for the prevention of arrhythmia and other cardiovascular diseases caused by COVID-19-induced myocardial injury.

The principal limitation of the current study is that we did not investigate the electrophysiological effects of NOB on human ventricular myocytes. Moreover, we did not investigate the potential interaction of NOB on the slow inactivation of the Na_V_1.5 channel and other ion channels which demonstrated the target specificity of NOB. These results should be further validated in human ventricular myocytes to confirm the therapeutic value of NOB as a cardio-protective agent and in treating ventricular arrhythmias.

## 4. Materials and Methods

### 4.1. Animals and Ethics Statement

The Yangzhou University Comparative Medical Center provided Sprague–Dawley (male and female) rats weighing 200 ± 20 g. The rats were maintained in a room of constant temperature and humidity (25 ± 2 °C and 55 ± 5%), with a reverse 12 h light/12 h dark cycle. The Institutional Animal Care and Ethics Committee of Yangzhou University approved the animal protocol (SCXK (su) 2022-0044), and all experimental procedures were executed in compliance with the guidelines and regulations.

### 4.2. Drugs and Reagents

NOB (≥99%) and aconitine (≥99%) were purchased from Chengdu EFA Biotechnology Co. Ltd. (Chengdu, China). Heparin sodium and glucose were obtained from Beijing Solarbio Science and Technology Co., Ltd. (Beijing, China). Collagenase II (Cat. no. 44N15308B) and egtazic acid EGTA (cat. no. 101257840) were obtained from the Worthington Company (Lakewood, NJ, USA) and Sigma (Aldrich, St. Louis, MO, USA), respectively. Taurine (cat. no. 102269839T), CsCl (cat. no. 101368097), TEACl (cat. no. 101394223), MgATP (cat. no. 1001571838), and HEPES (cat. no. 102394459) were purchased from Sigma Aldrich (St. Louis, MO, USA). Bovine serum albumin (BSA; cat. no. 8398F) and tetrodotoxin (TTX; cat. no. AF02) were provided by ICN (Santa Ana, CA, USA) and Affix Scientific Company (Kfar Yona, Israel), respectively.

### 4.3. Solutions

The Ca^2+^-free Tyrode’s solution (pH adjusted to 7.3 with 3 mol/L NaOH) consisted of the following ingredients (mmol/L): NaCl, 135; KCl, 5.4; MgCl_2_, 1.0; NaH_2_PO_4_, 0.33; HEPES, 5.0; and glucose, 10.0. To prepare the Ca^2+^-saturated Tyrode’s solution, 1.8 mmol/L CaCl_2_ was added to the Ca^2+^-free Tyrode’s solution.

The KB solution (pH adjusted to 7.4 with 3 mmol/L KOH) consisted of the following ingredients in mmol/L: 89.0 KOH, 25.0 KCl, 70.0 L-glutamate, 10.0 NaH_2_PO_4_, 10.0 taurine, 11.0 glucose, 5.0 HEPES, and 1.0 EGTA. 

The solution (pH adjusted to 7.4 with 99% CsOH) in the pipette used to record *I*_Na_ consisted of the following ingredients in mmol/L: 133.0 CsCl, 5.0 NaCl, 20.0 TEACl, 10.0 EGTA, 10.0 HEPES, and 5.0 MgATP.

The bath solution (adjusted to pH 7.4 with 3 mmol/L NaOH) consisted of the following ingredients in mmol/L: 135.0 NaCl, 5.4 CsCl, 1.0 MgCl_2_, 1.8 CaCl_2_, 5.0 HEPES, 10.0 glucose, and 0.1 CdCl_2_.

### 4.4. Establishment of Ventricular Arrhythmia In Vivo

The in vivo protocol for ventricular arrhythmia was based on an already existing protocol [46]. Thirty male rats were selected and fed for 3 days to ensure they had normal heart rates. The rats were divided into three parallel groups: the normal saline group (Saline group, *n* = 10), the NOB group (*n* = 10), and the amiodarone group (*n* = 10). Before the establishment of ventricular arrhythmia, anesthesia was induced by an intraperitoneal (i.p.) injection of chloral hydrate (0.38 g/kg). The rats were fixed in a supine position and a lead II electrocardiogram (ECG) was recorded simultaneously using an RM6240 Biological Data Acquisition and Analysis System (Chengdu Instrument Factory, Chengdu, China). After maintaining stable ECG recordings for 10 min, the rats in the three groups were intravenously injected with different solutions via the tail vein within 30 s: Saline group, 1.5 mL/kg saline; NOB group, 1.5 mL/kg saline containing NOB (10 mL/kg); and amiodarone group, 1.5 mL/kg saline containing amiodarone (5 mg/kg). After 10 min of stabilization, the rats were injected with aconitine (0.001%, 40 μg/kg) via the caudal vein. The ECGs of the three groups of rats were recorded for 1 h, focusing on the onset time of ventricular arrhythmia (i.e., ventricular premature (VP) and ventricular tachycardia (VT)) and the incidence of fatal ventricular fibrillation (VF).

### 4.5. Acute Single-Cell Isolation

Ventricular myocytes were isolated from Sprague–Dawley rats (male and female) weighing 200 ± 20 g, as previously described [47]. Ten minutes before anesthesia by i.p. injection with 2% sodium pentobarbital (40 mg/kg), the rats were anticoagulated by i.p. injection of heparin (2000 IU/kg). After successful anesthesia, the animals were fixed in a supine position, then quickly pinned and thoracotomized. The whole hearts were excised and rapidly immersed in Ca^2+^-free Tyrode’s solution at 4 °C. After slight trimming of the redundant tissue, the hearts were anchored in a Langendorff device by ligating the aorta. The remaining blood was discharged by 30 s of Ca^2+^-saturated perfusion and 10 min of Ca^2+^-free Tyrode’s solution. Finally, the hearts were digested for approximately 25 min in Ca^2+^-free Tyrode’s solution containing type II collagenase (0.4 g/L), BSA (1 g/L), and taurine (0.4 g/L) until they increased in size. The hearts were seen to soften, with thick, turbid fluid exudate. All perfusion and digestion processes were kept at 37 °C with 100% oxygen saturation. The ventricular tissue was then removed, dissected, and filtered through a 100-mesh filter to obtain single cells. The single cells were washed thrice and reserved in KB solution for 0.5 h of perfusion with oxygen.

### 4.6. Stimulus Protocols

A single, square-wave pulse stimulation protocol was used to instruct voltage for 30 ms at −30 mV to induce an inward current from a holding potential of −80 mV.

The stimulation protocol for the Na^+^ channel current-voltage curve (*I*–*V* curve) was as follows: the voltage clamp mode was sustained at 80 mV, and the single cells were stimulated with a series of square waves (−70 to 50 mV) with an amplitude step of 5 mV at 0.5 Hz for 30 ms.

The *I*_Na_ activation current stimulation protocol was as follows: the ventricular myocytes were stimulated by a series of square waves (−80 to 50 mV) at a step of 5 mV with a holding potential at 80 mV of 0.5 Hz, for 30 ms.

The detection protocol for the *I*_Na_ inactivation curve was a double-pulse method used at −80 mV in the voltage clamp mode. A pretreatment pulse was used to depolarize the cells (−140 to 40 mV) in 10 mV steps for 50 ms. Next, a −30-mV pulse was used to stimulate the ventricular cells for 25 ms.

A double-pulse stimulation protocol with a holding potential of −80 mV was used to test the recovery curve after *I*_Na_ inactivation. The cells were stimulated with a −30 mV wave pulse lasting 30 ms for depolarization. After a set interval, 30 mV stimulatory pulses were applied, lasting 25 ms. A total of 15 pulses were applied to the ventricular myocytes, with the interval between pulses incrementing by 10 ms.

### 4.7. Whole-Cell Patch-Clamp Recording

Cells for patch-clamp assessment are required to be long and rod-shaped with a smooth, intact cytomembrane and distinct horizontal striation without spontaneous contract vibration. The cells were transferred from the suspension to a Petri dish at normal temperature (20–25 °C). The cells were left to stand for 10 min to allow them to adhere to the dish, and the dish was filled with the extracellular solution to remove the remaining KB solution. The MP-225 Micromanipulator System (Sutter Company, Novato, CA, USA), EPC-10 USB/Patchmaster Single Channel Patch-clamp amplifier (HEKA Company, Nordrhein-Westfalen, Germany), and an IX73 inverted scientific microscope (Olympus Company, Nagano, Japan) were used in the whole-cell patch-clamp system. Borosilicate glass microelectrodes (outer diameter 1.50 mm, inner diameter 1.14 mm; Wuhan Microprobe Co., Ltd., Wuhan, China) with a resistance of 2–6 MΩ were pulled with a P-97 microelectrode puller (Sutter Company, CA, USA). The application and information gathering of the stimulation protocol were based on the Patchmaster program (version v2x73.5; HEKA Company, Nordrhein-Westfalen, Germany).

### 4.8. Molecular Modeling and Computational Methods

The crystallography structures of the human voltage-gated sodium channel Na_V_1.5 (PDB ID: 6LQA), the rat voltage-gate sodium channel Na_V_1.5 (PDB ID: 6UZ3), and open state structure of rNav1.5/QQQ (PDB ID: 7FBS) [32] were retrieved from the Research Collaboratory for Structural Bioinformatics (RCSB) Protein Data Bank. The chemical structures of the natural compounds were obtained from the PubChem compound database (http://pubchem.ncbi.nlm.nih.gov/ (accessed on 15 October 2022) with PubChem CIDs of 72344 (nobiletin), 245005 (aconitine), and 2157 (amiodarone). Then, molecular docking calculations were executed using AutoDock Vina software. For the molecular docking calculations, the pdbqt files for the proteins and ligands were prepared according to the AutoDock protocol. All docking parameters were conserved to their default values, except the maximum number of energy evaluations (eval) and the number of genetic algorithm (GA) runs. The docking grid was made as the binding site for the receptor with a grid size of 40 Å × 40 Å × 40 Å. The grid spacing value was adjusted to 0.375 Å. Gasteiger atomic partial charges were assigned for all investigated ligands. The PyMol (PyMOL Molecular Graphics System, version 1.7) program (https://pymol.org (accessed on 15 October 2022), was applied for visualization to obtain the hydrogen bond, hydrophobic, and electrostatic interactions [48].

The binding energies of the ligands with protein were calculated using the molecular mechanics/generalized Born surface area (MM-GBSA) approach with a GB model [20]. The binding energies (DG_binding_) were computed using the molecular docking complex, given by:

DG_binding_ = G_Complex_ − (G_ligand_ + G_receptor_) (5)

where the energy term (G) is estimated as:

G _x_ = E_vdw_ + E_ele_ + G_GB_ + G_SA_ (6)

with E_vdw_, E_ele_, G_GB_, and G_SA_ as the van der Waals, electrostatic, general Born solvation and surface area energies, respectively. For the inhibitors, entropy contributions were neglected.

### 4.9. Statistical Analysis

The data are expressed as the mean ± standard deviation (mean ± SD). Origin software (version 7.0; Microcal Software, Inc., Northampton, MA, USA) was used to analyze the experimental data. GraphPad Prism software (version 8.02, San Diego, CA, USA) was utilized to fit the curves. Two-tailed paired Student’s t-tests were used for comparisons of two means, analysis of variance (ANOVA) was used for the comparison of multiple means, and the χ^2^ test was used for the comparison of two incidences to assess the statistical significance.

## 5. Conclusions

In the present investigation, we preliminarily determined that NOB exerts an anti-arrhythmic effect by studying the electrophysiological effects and through molecular docking analysis. NOB exerts anti-arrhythmic effects by inhibiting *I*_Na_ and retarding the steady-state deactivation process. Thus, NOB is a potential option as a class *I*_Na_ channel blocker in cardio-protective administration and the treatment of arrhythmia.

## Figures and Tables

**Table 1:** Incidence of ventricular fibrillation occurring during 30 min of ECG recording. Within an observation of 30 min, 19 of 20 rats had ventricular fibrillation (VF) in the normal saline group (Saline), 2 of 20 rats had VF in the nobiletin group (NOB), and 1 of 20 rats had VF in the amiodarone group. (*n* = 20, ## *p* < 0.01 vs. Saline group, as assessed using a χ^2^ test).

Saline	NOB	Amiodarone
95%	10% ##	5% ##

**Table 2:** Peak current density at −40 mV measured for different concentrations of NOB and amiodarone.

Group (Concentration μmol/L)	Current Density (pA/pF)
Control	−87.79 ± 4.21
NOB (5)	−86.36 ± 4.63
NOB (10)	−81.30 ± 5.01 *
NOB (25)	−61.87 ± 2.54 **
NOB (50)	−50.88 ± 5.41 **
NOB (100)	−38.90 ± 3.21 ***
NOB (200)	−29.09 ± 2.85 ***
Amiodarone (24.24)	−27.34 ± 4.63 ***

Data are presented as means ± standard deviation (*n* = 10). pA/pF means membrane current/capacitance. * *p* < 0.05, ** *p* < 0.01, *** *p* < 0.001 vs. Control group.

**Table 3:** The binding affinity, MM-GBSA binding energy (kcal/mol), and detailed intermolecular binding interactions of the rat voltage-gated sodium channel (termed rNav1.5), open state structure of rNav1.5 (termed rNav1.5/QQQ), and human voltage-gated sodium channel (termed hNav1.5) with nobiletin in molecular simulation.

Ion Channel Proteins	Binding Affinity (kcal/mol)	MM-GBSA Binding Energy (kcal/mol)	π-π Stacking	Hydrogen Bonding	Hydrophobic Interaction
rNav1.5	−6.655	−36.44	Phe-1762	Ser-1712	Ile-1468, Leu-1464, Ser-1460, Phe-1420, Lys-1421
rNav1.5/QQQ	−6.562	−29.32	Phe-1762	Ser-1712	Ile-1468, Leu-1464, Ser-1460, Phe-1420, Lys-1421
hNa_v_1.5	−5.693	−51.71		Gln-371, Lys-1419, Ser-1458	Phe-1418, Leu-1462, Phe-1760

## Data Availability

The raw data supporting the conclusions of this article will be madeavailable by the corresponding authors upon reasonable request.

## Supplementary Materials

The supporting information can be downloaded at: https://www.mdpi.com/article/10.3390/ijms232315175/s1.
