# Supplementary material for: Inhibitory Effects of Nobiletin on Voltage-Gated Na+ Channel in Rat Ventricular Myocytes Based on Electrophysiological Analysis and Molecular Docking Method
Source: Int J Mol Sci. 2022 Dec 2;23(23):15175. doi: 10.3390/ijms232315175 (PMC9736168; doi:10.3390/ijms232315175)

Supplementary Table S1. The detailed relative binding-free energies (kcal/mol) of Nobiletin, Aconitine and Amiodarone binding with rNav1.5, rNav1.5/QQQ and hNav1.5 obtained by Prime MM–GBSA.

Ion channel proteins	Compounds	MMGBSA dG Bind	MMGBSA dG Bind Coulomb	MMGBSA dG Bind Covalent	MMGBSA dG Bind Hbond	MMGBSA dG Bind Lipo	MMGBSA dG Bind Packing	MMGBSA dG Bind SelfCont	MMGBSA dG Bind Solv GB	MMGBSA dG Bind vdW
rNav1.5	Nobiletin	-36.44	-3.99	2.35	-3.16	-8.21	-0.17	0.00	16.30	-39.57
rNav1.5/QQQ	Nobiletin	-29.32	-0.84	0.79	-3.91	-5.55	-0.13	0.00	14.65	-34.34
hNav1.5	Nobiletin	-51.71	-6.64	0.36	-0.63	-18.13	-0.71	0.00	21.90	-47.86
hNav1.5	Aconitine	-62.91	-8.55	3.78	-12.99	-13.18	-1.30	0.00	18.68	-49.35
hNav1.5	Amiodarone	-42.26	-8.71	5.45	-5.38	-12.25	-0.98	0.00	20.09	-40.48

Supplementary Figure S1. Computer modeling of active sites regarding the potential key target of Aconitine (A) and Amiodarone (B) in the active domain of human voltage-gated sodium channel (termed hNav1.5). The residues of ligand bind domain (LBD) proteins representing hydrogen bonds are depicted and annotated as yellow dotted lines.

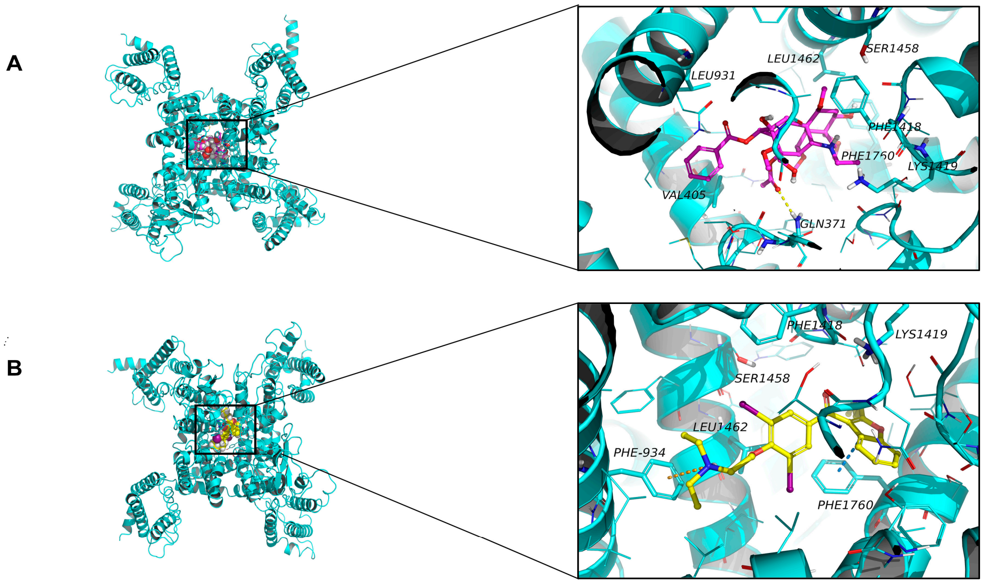

Supplementary Table S2. The detailed intermolecular binding interactions of human voltage-gated sodium channel (termed hNav1.5) with Aconitine and Amiodarone in molecular simulation.

Compounds	Binding affinity (kcal/mol)	π stacking	Hydrogen bonding	Hydrophobic interaction
Aconitine	-8.889		Gln-371	Val-405, Leu-931, Phe-1418, Lys-1419, Ser-1458, Leu-1462, Phe-1760
Amiodarone	-6.465	Phe-934 [1]<br>Phe-1760 [2]		Phe-1418, Lys-1419, Ser-1458, Leu-1462

[1] Amiodarone formed cation-π stacking with Phe-934 when docking with hNav1.5 .

[2] Amiodarone formed π-π stacking with Phe-1760 when docking with hNav1.5.

Supplementary Figure S2. The sequence alignment of rNav1.5 (Uniprot ID: P15389) and hNav1.5 (Uniprot ID: Q14524). The conserved amino acid residues are colored in red and shaded in yellow. The similar amino acid residues are shaded in green. The voltage-sensing domain (VSD) I, II, III and IV are squared in red, dark blue, pink and black, respectively.

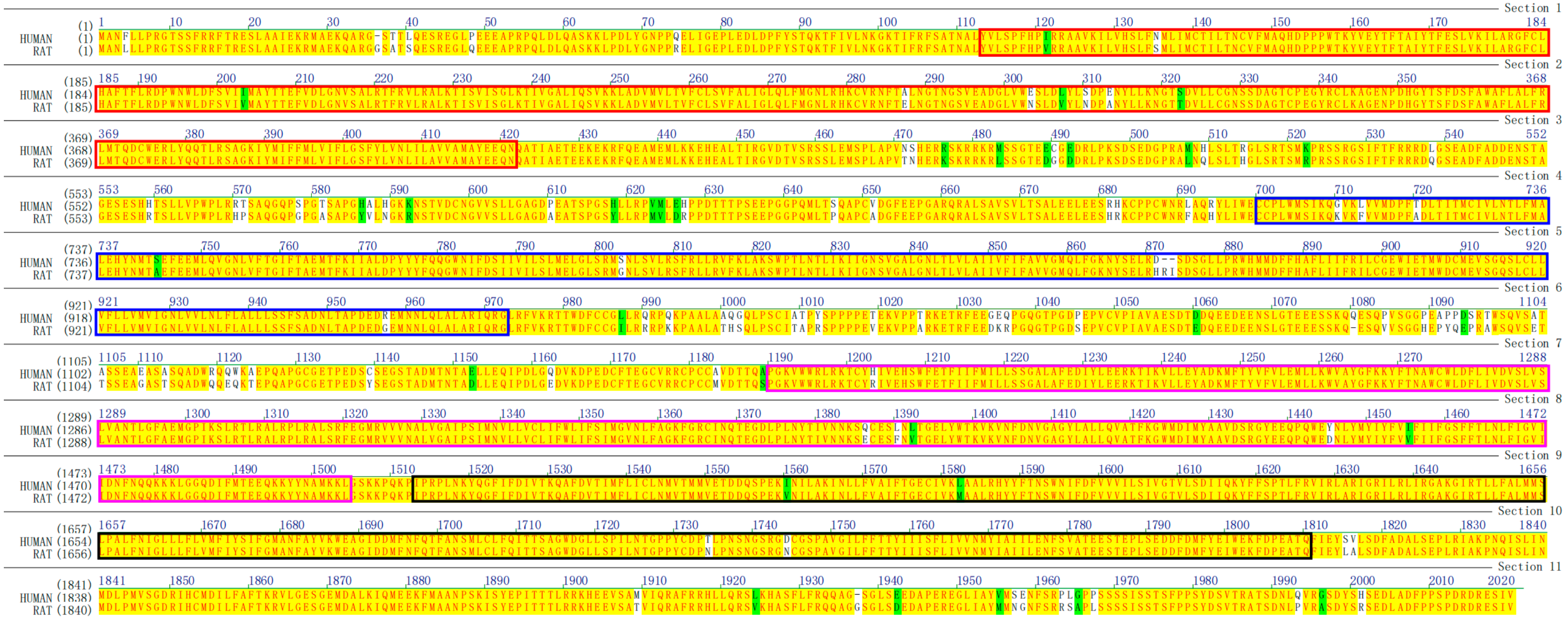

Supplement: Supplementary file 1 [file ijms-23-15175-s001.zip › ijms-2042292-supplementary.pdf]
